# Autophagy-related gene LAPTM4B promotes the progression of renal clear cell carcinoma and is associated with immunity

**DOI:** 10.3389/fphar.2023.1118217

**Published:** 2023-03-02

**Authors:** He Wang, Qibo Wang, Yaoyao Wu, Jianmin Lou, Shaoxing Zhu, Yipeng Xu

**Affiliations:** ^1^ The Second Clinical Medical College, Zhejiang Chinese Medical University, Hangzhou, China; ^2^ Department of Urology, Cancer Hospital of University of Chinese Academy of Sciences (Zhejiang Cancer Hospital), Hangzhou, China; ^3^ Department of Urology, Fujian Medical University Union Hospital, Fuzhou, China

**Keywords:** renal cell carcinoma, ccRCC, autophagy, LAPTM4B, immune, cell proliferation, single-cell RNA sequencing

## Abstract

Renal cell carcinoma (RCC) is a common urologic disease. Currently, surgery is the primary treatment for renal cancer; immunotherapy is not as effective a treatment strategy as expected. Hence, understanding the mechanism in the tumor immune microenvironment (TME) and exploring novel immunotherapeutic targets are considered important. Recent studies have demonstrated that autophagy could affect the immune environment of renal cell carcinoma and induce proliferation and apoptosis of cancer cells. By comparing lysosomal genes and regulating autophagy genes, we identified the LAPTM4B gene to be related to RCC autophagy. By analyzing the TCGA-KIRC cohort using bioinformatics, we found M2 macrophages associated with tumor metastasis to be significantly increased in the immune microenvironment of patients with high expression of LAPTM4B. GO/KEGG/GSEA/GSVA results showed significant differences in tumor autophagy- and metastasis-related pathways. Single-cell sequencing was used to compare the expression of LAPTM4B in different cell types and obtain the differences in lysosomal and autophagy pathway activities in different ccRCC cells. Subsequently, we confirmed the differential expression of LAPTM4B in renal cell carcinoma of different Fuhrman grades using western blotting. Downregulation of LAPTM4B expression significantly reduced the proliferation of renal cell carcinoma cells and promoted cell apoptosis through cell experiments. Overall, our study demonstrated that the autophagy-related gene LAPTM4B plays a critical role in the TME of RCC, and suggested that LAPTM4B is a potential therapeutic target for RCC immunotherapy.

## 1 Introduction

Renal cell carcinoma (RCC) is one of the ten most common cancers in the United States, and RCC accounts for 90% of all kidney cancers. Statistics suggests that the RCC mortality accounts for 2% of all cancer deaths worldwide (Gray and Harris, 2019; Siegel et al., 2022), and the incidence of kidney cancer continues to be on the rise (Capitanio et al., 2019). According to the pathological classification, renal clear cell carcinoma (ccRCC) accounts for approximately 70%–80% of renal carcinomas (Rini et al., 2009). Treatment of metastatic ccRCC is still limited, and its prognosis is poor, with less than 10% of patients surviving after 5 years of diagnosis (Turajlic et al., 2018).

Tumor metastasis is the leading cause of death in patients with cancer (Maishi and Hida, 2017). The mechanism of tumor metastasis is a complex and diverse process, involving changes in the tumor microenvironment, metabolic changes, and other biological processes (López-Sánchez et al., 2020; Chen et al., 2021; Reticker-Flynn et al., 2022). Autophagy is a comprehensive process which includes intracellular degradation of senescent or dysfunctional organelles that are eventually transported to lysosomes for metabolism and excretion (Klionsky and Emr, 2000; Mizushima and Levine, 2020). The lysosome is an autophagy organelle and an essential mediator of the catabolic process (Settembre et al., 2011; Buratta et al., 2020). Autophagy works as a crucial role in the tumors cell goes, since it can inhibit tumor growth in early stages of tumors and promote the occurrence and metastasis of cancer in late stages of tumors (Choi, 2012). Previous studies had shown enhanced STAT1/STAT6 phosphorylation (pSTAT1/pSTAT6)-dependent autophagy can suppress the proliferation and invasion of RCC cells (Chai et al., 2022). The induction of autophagy can also help RCC cells resist the killing effect of immune cells, which is an essential reason for the unsatisfactory effect of immunotherapy in RCC (Messai et al., 2014). Therefore, understanding of the specific mechanism of autophagy in the occurrence and metastasis of RCC will help in the intervention and improve the prognosis of patients with RCC early (Pasquier, 2015; Li et al., 2021).

Lysosomal protein transmembrane 4 beta (LAPTM4B) is a protein-coding gene required for the function of lysosomes (Vergarajauregui et al., 2011). It activates the mTORC1 signaling pathway (Tan et al., 2015a) and acts as a negative regulator of TGFB1 in regulatory T cells (Huygens et al., 2015), thereby participating in the cell death program (Blom et al., 2015). Besides, LAPTM4B is generally highly expressed in solid tumors, can promote autophagy and tolerance to metabolic stress in cancer cells, and is an essential gene for adjuvant drug resistance (Li et al., 2011; Li et al., 2012; Meng et al., 2016). However, the role of LAPTM4B in RCC, especially in the specific mechanism of RCC proliferation and metastasis, remains unclear. In this study, using the KEGG database and literature review, we identified the gene sets related to the lysosomal pathway and those regulating the autophagy pathway. With the help of bioinformatics, we identified the autophagy-related gene LAPTM4B and observed the prognosis of LAPTM4B in ccRCC. The immunological and pathway differences in the LAPTM4B-expression groups were analyzed using the GO/KEGG/GSVA method. Finally, the effects of LAPTM4B on the proliferation, apoptosis, and metastasis of tumor cells in ccRCC were verified using cell experiments.

## 2 Materials and methods

### 2.1 Data acquisition and analysis

Clinical information and RNA-seq of the TCGA-KIRC cohort (534 kidney tumor tissues, 72 normal kidney tissues) were derived from the UCSC Xena database (http://xena.ucsc.edu/). Lysosomal gene set (121 genes) and autophagy gene set (36 genes) could be obtained from the GSEA database (https://www.gsea-msigdb.org/gsea/msigdb/index.jsp) and from the literature (Li et al., 2011). Single-cell ccRCC chips (GSM4630028 and GSM4630029) were obtained from the GEO database (https://www.ncbi.nlm.nih.gov/geo/).

### 2.2 GO/KEGG/GSEA/GSVA functional enrichment

A total of 534 tumor tissues were divided into low- and high-expression groups in line with the median expression of LAPTM4B. The low group represented low expression of LAPTM4B based on the median value, and the high group represented high expression of the same. A total of 209 differential genes (DEGs) were obtained using the “limma” package, and the filtering value considered fold change >1.2 and *p* < 0.05. To evaluate the biological function between different groups or across patients with ccRCC, DEGs were analyzed for GO/KEGG/GSEA enrichment using the “clusterProfile” package. Thereafter, GSVA enrichment analysis was performed using the “GSVA” R package, which evaluated the difference in biological pathway activity between the two groups. For GSVA analysis, “h.all.v2022.1. Hs symbols. gmt” and “c2. cp.kegg.v2022.1. Hs. symbols.gmt” were downloaded from the MSigDB database (https://www.gsea-msigdb.org/gsea/msigdb/index.jsp), and significantly enriched pathways were filtered using an adjusted *p*-value <0.05.

### 2.3 Immune infiltrates and immune checkpoints

Single-sample gene set enrichment analysis (ssGSEA) is a GSEA for a single sample. Ranking of the gene list and ES calculation depend on the expression value of genes in the sample rather than on the correlation between genes and phenotypes (Bindea et al., 2013). The “CIBERSORT” algorithm makes a deconvolution method extract features from single-cell RNA-seq data and inversely calculates the proportion of various cellular components in bulk-seq (Finotello and Trajanoski, 2018). The ssGSEA method was used to score the immune abundance of 534 tumor samples, and “CIBERSORT” was used to evaluate the abundance of immune cells in the 534 tumor samples. Immune differences between the different groups were determined according to the immune cell abundance scores. Pearson’s statistical method was applied to compare the correlation between LAPTM4B and immune cells, and all samples had a *p*-value <0.05. To compare the direct relationship between LAPMTM4B and immunotherapy, we calculated the correlation between LAPTM4B and immune checkpoint genes (Postow et al., 2018; Hu et al., 2021).

### 2.4 Evaluation of the pathway activity in single cells of ccRCC

The filter values were set as follows: nFeature RNA >300 and percent. mt < 10 and percent HB < 0.1; quality control was performed on GSM4630028 and GSM4630029, and the “harmony” package was used to integrate the samples when periodic genes did not affect the data. We got 14 clusters annotated with the “SingleR” package to get 8 cell types. The “AUCell” and “clusterProfiler” packages were used to calculate the activities of lysosomal and autophagy pathways in different groups. Pathway activation was calculated based on “c2.cp.kegg.v2022.1. Hs.symbols.gmt”, which is available in the MSigDB database.

### 2.5 Cell culture

Two human ccRCC cell lines were used in this study (786-O and ACHN). All the cell lines were purchased from the National Collection of Authenticated Cell Cultures (Shanghai, China). Cells were incubated at 37°C with 5% CO_2_ in a humidified atmosphere, with the composition of culture medium as follows: RPMI-1640 medium (Gibco, Gaithersburg, MD, United States) supplemented with 10% FBS (Gibco, United States) and 1% penicillin/streptomycin (PS).

### 2.6 Vector constructs, lentivirus production, and cell transfection

Short hairpin RNAs (shRNA) were packaged into a lentivirus (LV). Shanghai Genechem commercially constructed the LV-LAPTM4B-shRNA (shLAPTM4B). The empty lentiviral construct, shCtrl, was used as a negative control. The lentiviral vectors were used at an appropriate multiplicity of infection (MOI) to infect 786-O and ACHN cells using enhanced infection solution (Shanghai Genechem Co., Ltd.). The transfection efficiency and expression of LAPTM4B were analyzed using quantitative RT-PCR (qRT-PCR) and western blotting.

### 2.7 qRT-PCR

Total RNA was extracted by TRIzol reagent (Invitrogen, United States) and then reverse-transcribed into cDNA using the PrimeScript™ 1st strand cDNA synthesis kit (Takara, Japan), according to the manufacturer’s protocol. The cDNA concentration was quantified using a cell Imaging Multi-mode Reader (BioTek Instruments, Inc.). After the cDNA was mixed with SYBR Green (Bio-Rad, United States), quantitative real-time PCR was performed using the CFX96 Touch™ Real-Time PCR Detection System (Bio-Rad), with the housekeeping gene GAPDH as an internal control. The thermocycling parameters were as follows: 95°C for 2 min, followed by 40 cycles of 95°C for 20 s, 58°C for 20 s, and 72°C for 15 s; from 65°C to 95°C; and an increase of 0.5°C every 5 s. The ΔCt method was used to calculate the relative mRNA transcript abundance. Nucleotide sequences of the primers used are as follows:

LAPTM4B-F: 5ʹ-TCA​ATG​CTG​TGG​TAC​TGT​TGA​TT-3ʹ.

LAPTM4B-R: 5ʹ-GTA​CGC​TCC​GTA​AGT​AGC​CAT​A-3ʹ.

GAPDH-F: 5ʹ-TGA​CTT​CAA​CAG​CGA​CAC​CCA-3ʹ.

GAPDH-R: 5ʹ-CAC​CCT​GTT​GCT​GTA​GCC​AAA-3ʹ.

### 2.8 Western blotting

Expression of LAPTM4B was analyzed using western blotting. Total cell and tissue lysates were prepared in RIPA lysis buffer containing phenylmethylsulfonyl fluoride (PMSF). Protein concentration was determined using a BCA protein assay kit (Beyotime, China). Equal quantities of proteins were separated on SDS-PAGE gels and transferred onto polyvinylidene difluoride membranes (Millipore). The membranes were blocked with 5% nonfat milk for 1 h at temperature, and incubated with primary antibodies overnight at 4°C. The primary antibodies used were anti-LAPTM4B (F1804, Sigma) and anti-GAPDH (sc-32233, Santa Cruz). Goat anti-rabbit IgG (H + L)-HRP (1:3,000; Bio-Rad Laboratories, Inc.) was used as the secondary antibody and was incubated with the membrane at room temperature for 2 h. Protein bands were visualized using Clarity™ Western ECL Substrate (Bio-Rad Laboratories, Inc.).

### 2.9 Cell proliferation assay

ccRCC cells were plated in 96-well plates at a density of 3000 cells/well after infection with shRNA lentiviral vector for 3 days. Cell proliferation was continuously detected using a Celigo Image Cytometer for 5 days.

### 2.10 MTT assay

MTT assay was performed to assess cell viability, according to the manufacturer’s instructions. After infection with shRNA lentivirus for 3 days, the cells were seeded at a density of 3000 cells/well in 96-well plates. Cell viability was detected continuously for 5 days. After incubation at different time points, ccRCC cells were incubated with MTT solution for 4 h and dissolved in 150 μL of dimethyl sulfoxide (DMSO). Cell viability was measured by scanning with a Cell Imaging Multi-mode Reader (BioTek) using a 490 nm filter.

### 2.11 Cell cycle analysis

Cell cycle distribution of ACHN and 786-O cells was analyzed through flow cytometry after infection with shRNA lentivirus for 5 days. Then the cells were harvested, washed, and fixed in 75% ice-cold ethanol overnight at −20°C. The cells were then rewashed, stained with PI/RNase (0.5 mL/test, 1 × 106 cells) (BD Pharmingen, United States), and incubated in the dark at room temperature for 15 min before being analyzed by flow cytometry (Guava Technologies; Merck KGaA, Germany). Data were analyzed using ModFit DNA analysis program (Windows version 4.0; Verity Software House).

### 2.12 Cell apoptosis analysis

Cell apoptosis was detected using an Annexin-V staining kit (BD Pharmingen, San Diego, CA, United States) after infection with shRNA lentivirus for 5 days. After incubation, ccRCC cells were harvested, stained with Annexin-V, and analyzed using a Guava Easy Cytometer (Guava Technologies; Merck KGaA, Darmstadt, Germany).

### 2.13 Statistical analysis

GraphPad Prism (9.0) and R (4.2.0) were used to perform statistical analyses. Pearson correlation analysis was adopted to compare the correlation of LAPTM4B expression with immune cells and immune checkpoint genes; the *t*-test and Kruskal-Wallis test were used for comparison between the groups. Log-rank test was used to compare the differences in survival viability between the two groups. *p* < 0.05 indicated significance (*, *p* < 0.05; **, *p* < 0.01; ***, *p* < 0.001; ****, *p* < 0.0001; ns, no significance).

## 3 Results

### 3.1 Expression and prognostic value of LAPTM4B in ccRCC tissue

**FIGURE 1:**
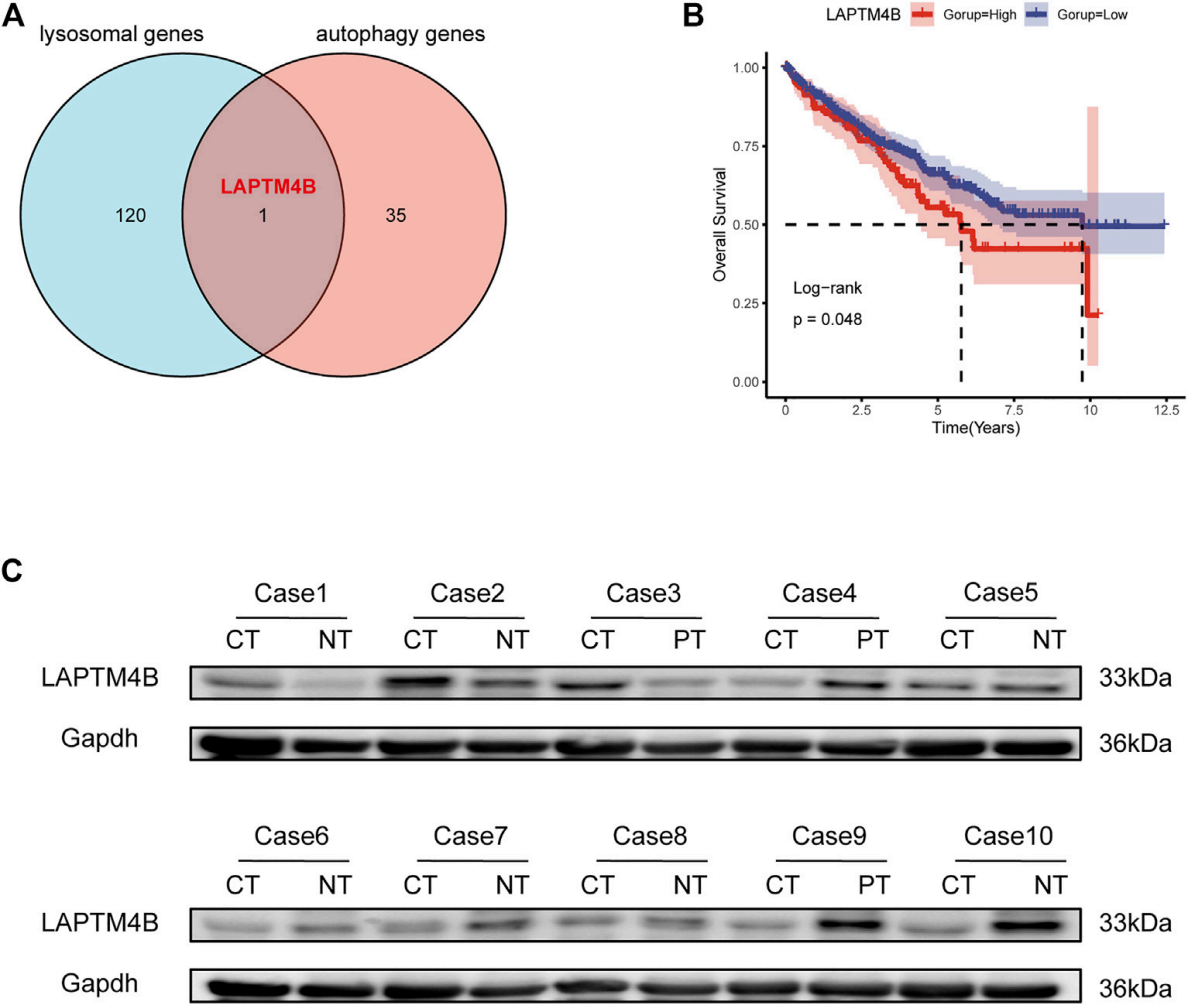
LAPTM4B expression and its prognostic value in ccRCC tissues. **(A)** The Venn diagram shows the common gene compared to the gene sets of the lysosomal and the regulating autophagy. **(B)** Log-Rank analysis shows that the overall survival of ccRCC (534 tumor samples) with high expression of LAPTM4B mRNA is worse; the group cut-off value is the upper quartile of LAPTM4B mRNA expression. **(C)** Western blot retrospectively verified the expression of LAPTM4B in 10 RCC patients. The expression of LAPTM4B in RCC tissues with Fuhrman grade Ⅳ is higher than that in the corresponding paracellular kidney tissues (Case 1-3). However, the expression of LAPTM4B in RCC tissues with Fuhrman grade Ⅰ is lower than that in adjacent renal tissues (Case 4-10) (CT: cancer tissues; NT: normal kidney tissue; PT: adjacent renal tissue).

The lysosomal gene set (121 genes) and autophagy gene set (36 genes) were obtained from the KEGG database as well as from published literature. To compare the lysosomal and autophagy genes, we obtained the common gene LAPTM4B (Figure 1A). We analyzed the correlation between LAPTM4B expression level and survival rate based on 534 patients from TCGA, and found that patients with low LAPTM4B expression levels had better overall survival than those with high LAPTM4B expression levels (Figure 1B).

Yang et al. (2018) had confirmed that LAPTM4B expression in tumor tissues is higher than in normal tissues. In this study, LAPTM4B content was detected using the urine survivin method, and was used to construct a diagnostic model of renal cell carcinoma with an AUC of 0.920. However, the specific mechanism of LAPTM4B in ccRCC was not discussed further in this study, although it is our study’s compensation and research focus. To explore the expression of LAPTM4B in different grades of ccRCC, based on western blotting, the protein levels of LAPTM4B in ccRCC tissues and matched normal kidney tissues from 10 patients (Fuhrman III/IV: 3 patients, Fuhrman I/II: 7 patients) were analyzed. Interestingly, results indicated the protein level of LAPTM4B to be higher in Fuhrman III/IV ccRCC tissues than in matched normal kidney tissues (Figure 1C). In comparison, the protein level of LAPTM4B was lower in Fuhrman I/II ccRCC tissues than in matched normal kidney tissues.

### 3.2 Functional enrichment

**FIGURE 2:**
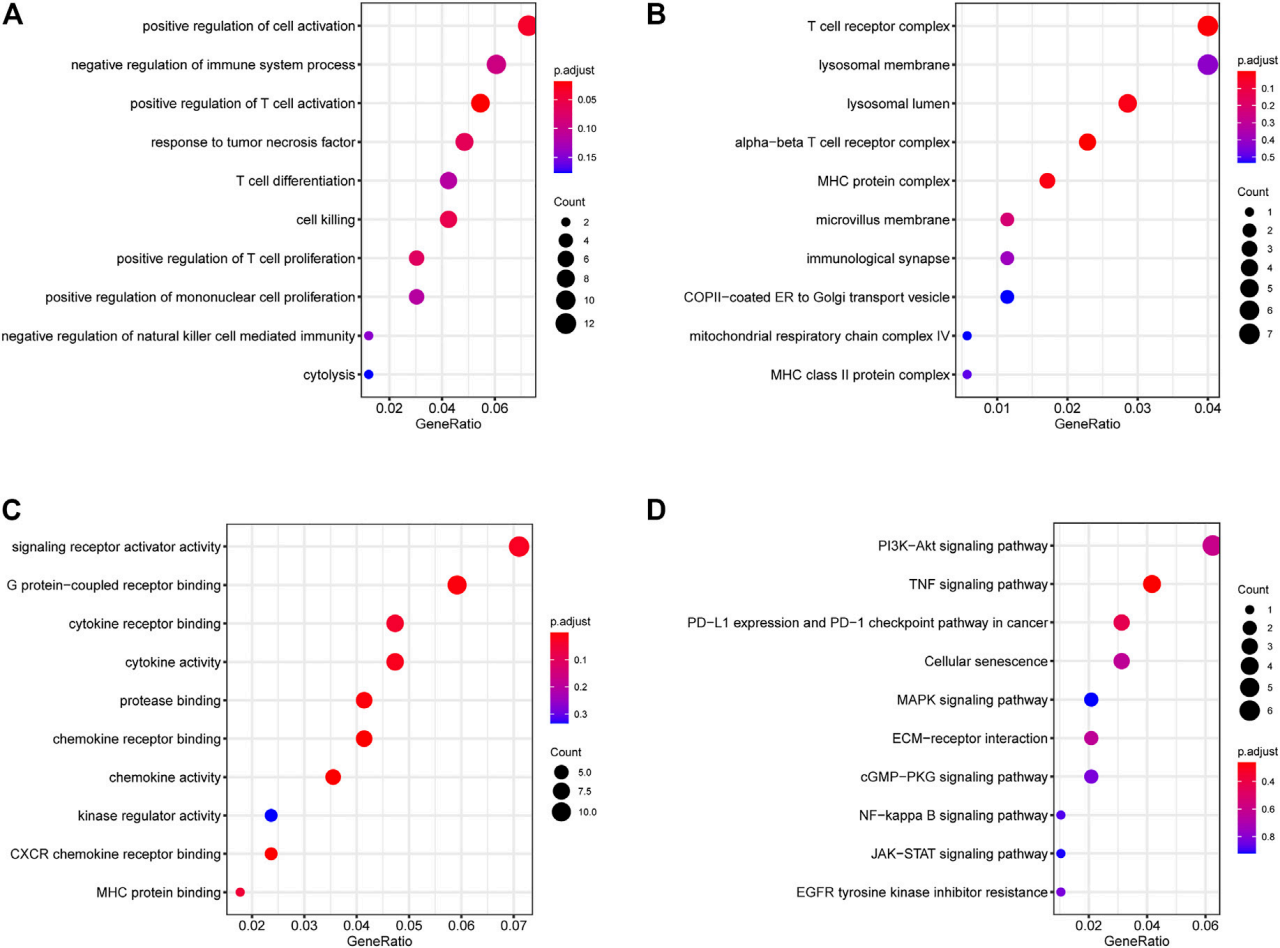
GO/KEGG functional enrichment of DEGs. **(A)** BP (biological process) **(B)** MF (molecular function) **(C)** CC (cellular component) **(D)** KEGG (Kyoto Encyclopedia of Genes and Genomes) functional enrichment. Signaling pathways related to immunity, autophagy and proliferation.

**FIGURE 3:**
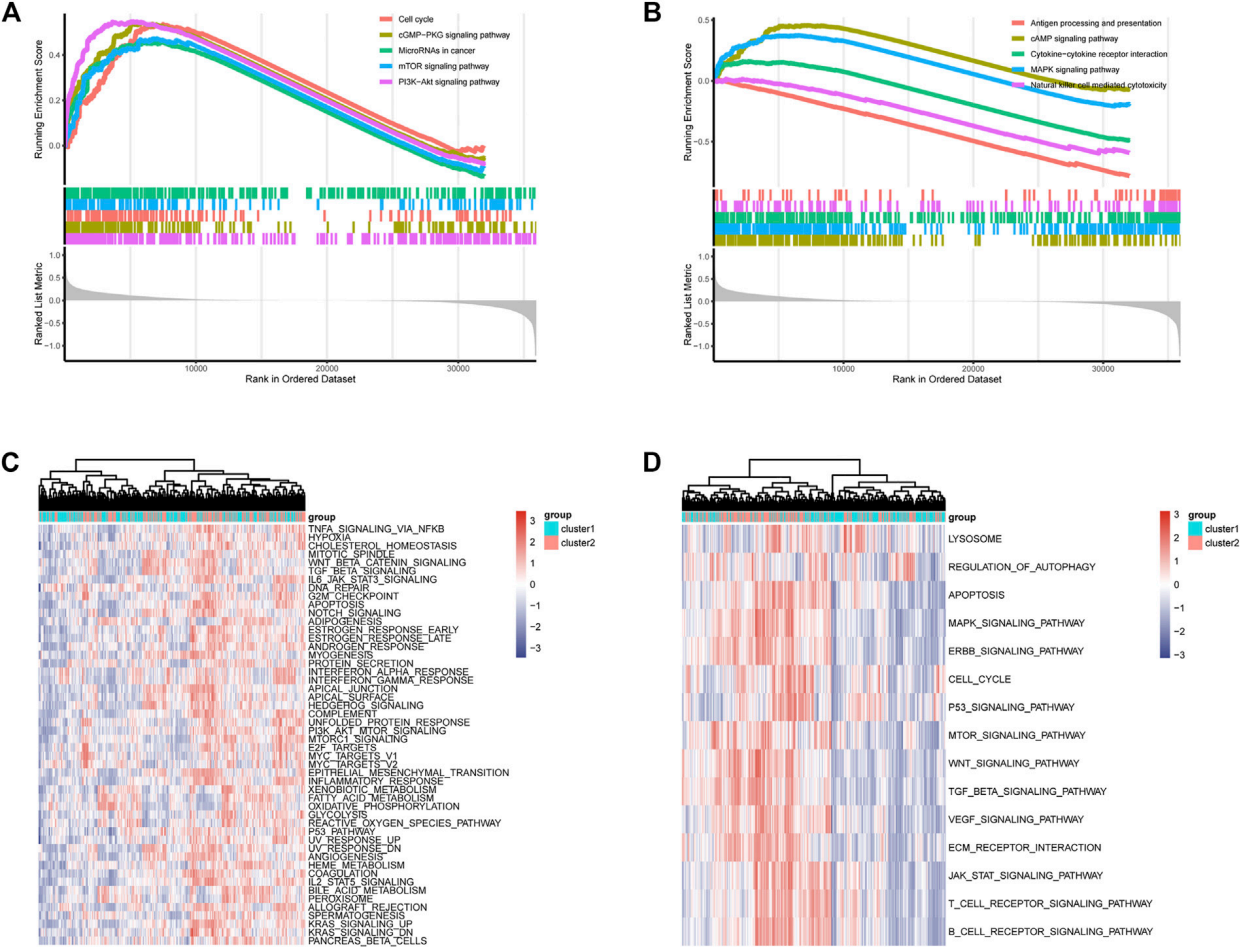
GSEA functional enrichment of DEGs and GSVA functional enrichment of two groups. **(A,B)** GSEA of DEGs, signaling pathways related to immunity, autophagy and proliferation. **(C)** GSVA (Gene Set Variation Analysis) showed the hallmark pathways differences between two groups. **(D)** GSVA showed differences in autophagy and proliferation-related pathways between two groups.

**FIGURE 4:**
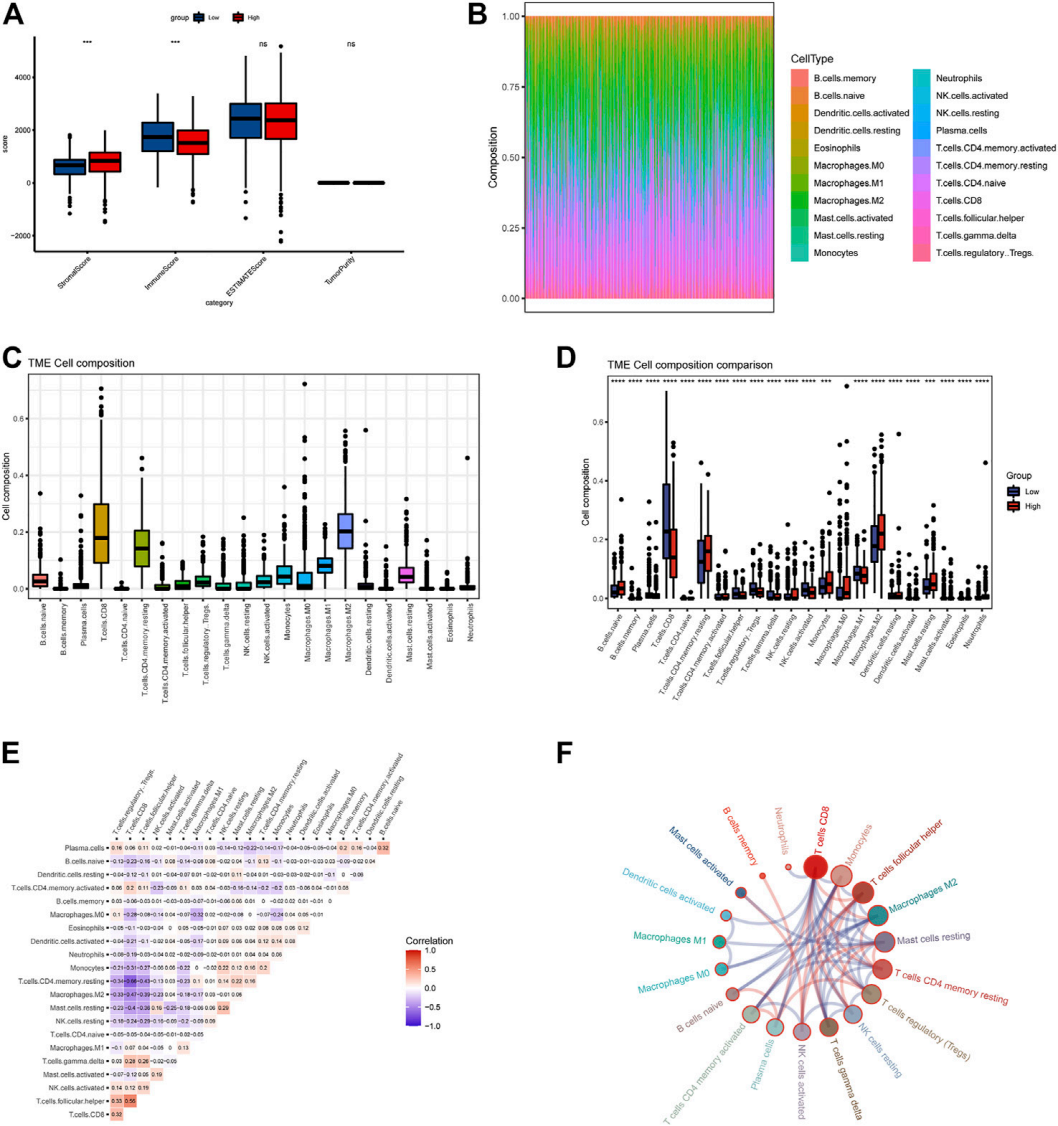
Evaluation of immune cell matrix and abundance of 534 TCGA tumor samples. **(A)** ssGSEA demonstrates differences in immune matrix scores between two groups. **(B,C)** Immune cells components between two groups by CIBERSORT method. **(D)** Differences in immune cell expression values between two groups. **(E,F)** The correlation between immune cells, all samples with a *p*-value of <0.05 were included. Red color means positive correlation, and blue represents negative correlation.

Functional enrichment results showed that the DEGs can participate in cell proliferation and death-related communication, and regulate both immune-related and checkpoint PD-L1-related pathways (Figure 2). According to the GSEA analysis results, the DEGs could participate in cell proliferation, death, cell cycle, and other pathways (Figures 3A, B). To further compare the activity differences in cell signaling pathways between the two groups, GSVA results of the hallmark gene set showed that TNFA-NKFB, JAK-STAT3, P53, and other pathways related to cancer cell proliferation and division were significantly increased (Figure 4C). KEGG-GSVA enrichment results also confirmed that the activities of pathways related to cancer cell proliferation and metastasis in the high group, such as the cell cycle, mTOR, VEGF, and JAK-STAT signaling pathways, were significantly increased (Figure 4D).

### 3.3 Quantitative evaluation of immune infiltration and checkpoints

**FIGURE 5:**
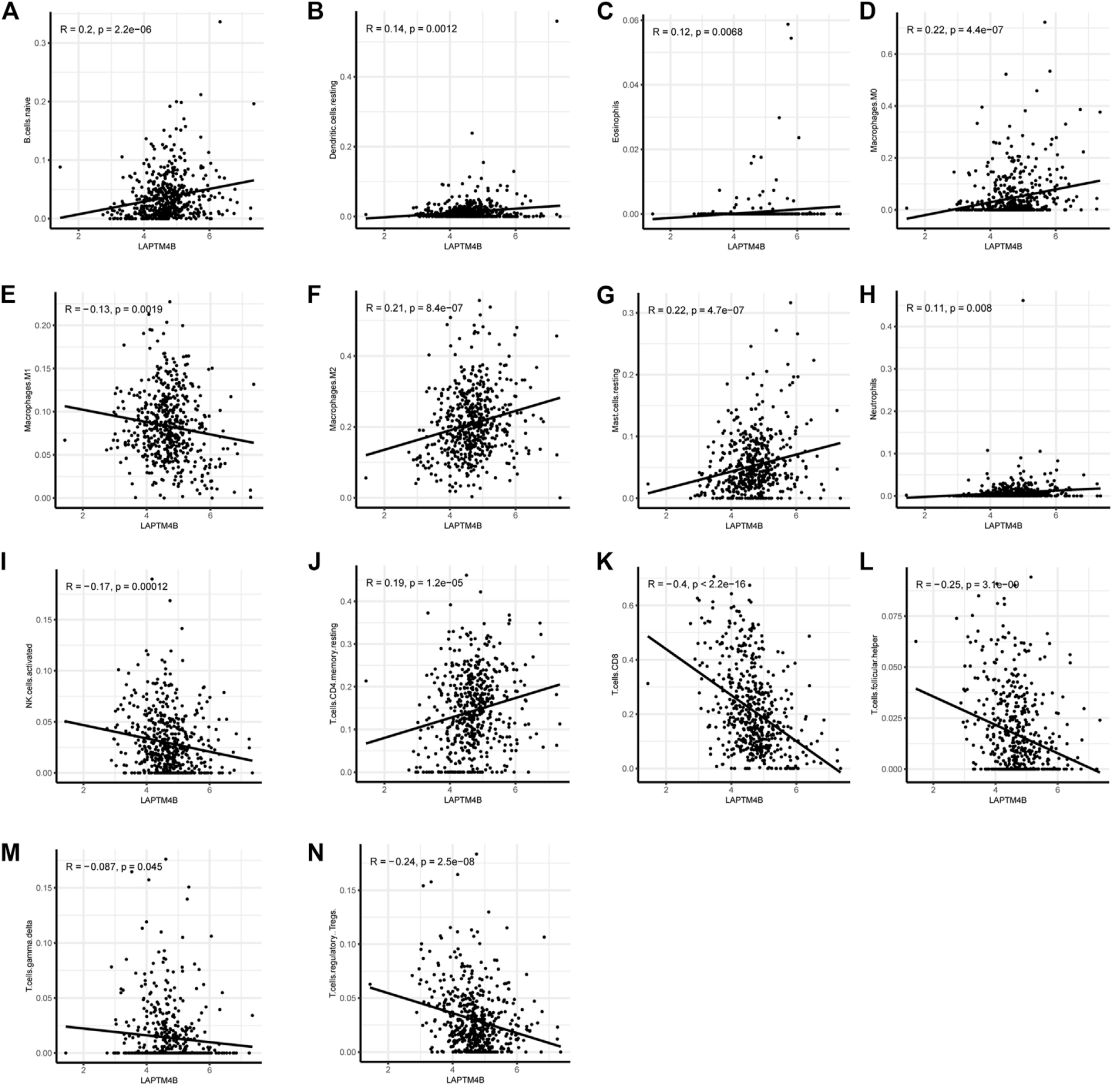
Correlation of LAPTM4B and immune cells. Correlation between LAPTM4B and various immune cells, *p* < 0.05 is the inclusion condition.

Based on the ssGSEA immune matrix algorithm, we observed a difference in the immune microenvironment score between the high-LAPTM4B expression and low-LAPTM4B expression groups. Stromal score of the high-group tumor tissues was higher than that of the low-group (*p* < 0.001). Immune score environment of the high-group tumor tissues was worse than that of the other tumor tissues (*p* < 001). However, there was no significant difference in the estimated score and tumor purity in two groups (Figure 4A). To further explore the specific immune cell differences in two groups, an estimation algorithm was used to evaluate the immune components of the two groups. Figures 4B, C shows the components and proportions of immune cells in 22 of the two groups. Comparison of the groups (Figure 4D) showed significant differences in immune cells between the two groups, with CD8^+^ T cells being significantly higher in the low group than in the high group, whereas M2 macrophages related to cancer cell proliferation and metastasis were significantly higher in the high group than in the low group. Results of immune cell correlation study showed (Figures 4E, F) a significant negative correlation between CD8^+^ T cells and M2 macrophages. Correlation analysis between LAPTM4B and immune cells showed a correlation between LAPTM and each immune cell (Figure 5); LAPTM4B was positively correlated with M2 macrophages associated with tumor metastasis. Correlation analysis between LAPTM4B and immune checkpoint genes showed that CD44 was positively correlated with VTCN1, TNFSF15, CD276, and CD44, with statistical significance, whereas the rest were negatively correlated (Table 1).

**TABLE 1:** Correlation between LAPTM4B and immune checkpoint genes.

	Cor	P-value		Cor	*P*-value
LAPTM4B	1	0	CD276	0.20170091	2.62E-06
TNFRSF14	−0.5078042	2.39E-36	CD200R1	−0.2016316	2.64E-06
CD27	−0.3820585	5.30E-20	CD44	0.19953749	3.37E-06
HHLA2	−0.3794512	9.89E-20	IDO2	−0.1988701	3.64E-06
PDCD1	−0.3776594	1.51E-19	LAIR1	−0.1982592	3.90E-06
LAG3	−0.3757836	2.36E-19	TNFRSF4	−0.1881799	1.20E-05
CD70	−0.3693446	1.06E-18	CD40	−0.1715308	6.77E-05
TNFRSF25	−0.3304269	4.54E-15	CD40LG	−0.1624319	0.00016337
VTCN1	0.3188382	4.40E-14	CD200	−0.1410375	0.00108361
TNFSF9	−0.3053486	5.49E-13	BTLA	−0.139387	0.00124117
CD244	−0.3046662	6.22E-13	TMIGD2	−0.1242578	0.00402969
CD48	−0.2970167	2.45E-12	CD86	−0.1204368	0.0053242
TNFSF15	0.29556494	3.16E-12	CD160	−0.1009671	0.01961192
IDO1	−0.2891279	9.65E-12	TNFSF18	0.08596206	0.04709214
LGALS9	−0.2762578	8.26E-11	NRP1	0.07088263	0.10179623
TIGIT	−0.2755401	9.28E-11	BTNL2	0.06691847	0.12246986
CTLA4	−0.2742933	1.14E-10	CD28	−0.0302603	0.48530928
HAVCR2	−0.2653485	4.68E-10	PDCD1LG2	0.01717442	0.69212484
TNFRSF9	−0.2592631	1.19E-09	CD80	−0.014326	0.74117852
C10orf54	−0.2546089	2.39E-09	CD274	0.01333529	0.75850141
TNFRSF18	−0.2296652	8.02E-08	ICOSLG	0.01315585	0.76165217
ADORA2A	−0.2270931	1.13E-07	TNFRSF8	−0.0097089	0.82288352
ICOS	−0.2225458	2.04E-07	KIR3DL1	−0.0068339	0.87480882
TNFSF14	−0.2136535	6.25E-07	TNFSF4	0.00577343	0.89411156

### 3.4 Evaluation of the pathway activity in single cells of ccRCC

**FIGURE 6:**
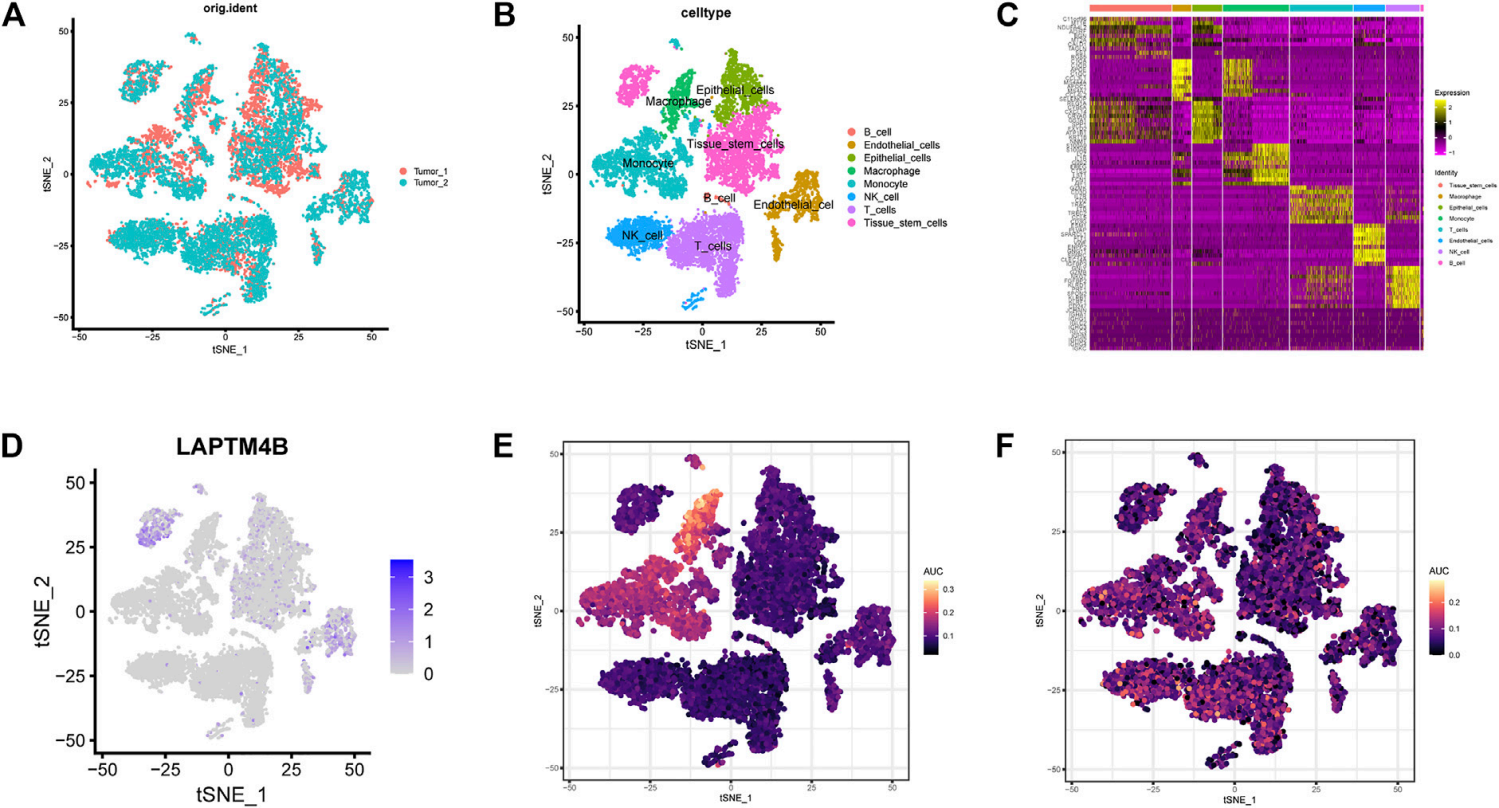
Evaluation of single cell pathway activity. **(A)** The tSNE diagram shows the results of integrating two ccRCC single-cell data using the harmony method. **(B)** The samples were annotated using the SingleR method, and 8 cell types were obtained. **(C)** The heatmap shows the mark genes for the 8 cell types. **(D)** Expression of LAPTM4B in eight celltypes. **(E)** Lysosomal pathway activity in each celltypes. **(F)** Autophagy pathway activity in each celltypes.

Harmony integration was performed on single-cell RNA sequencing samples of the two ccRCCs (Figure 6A), and PCA, tSNE, and UMAP methods were used for dimensionality reduction and clustering. The default parameter (logfc.threshold = 0.25) was used to identify the differential marker genes in each cluster (Figure 6C). After annotation using the SingleR algorithm, 8 cell types were obtained (Figures 6B, C). LAPTM4B was mainly expressed in stem, endothelial, and epithelial cells (Figure 6D). Results of the AUC activity algorithm based on the KEGG gene set showed that activity of the lysosomal pathway was higher in macrophages and monocytes than in other subsets (Figure 6E). However, the activity of autophagy-related pathways was relatively higher in NK cells, T cells, and monocytes (Figure 6F).

### 3.5 Knockdown of LAPTM4B inhibited the growth of RCC

**FIGURE 7:**
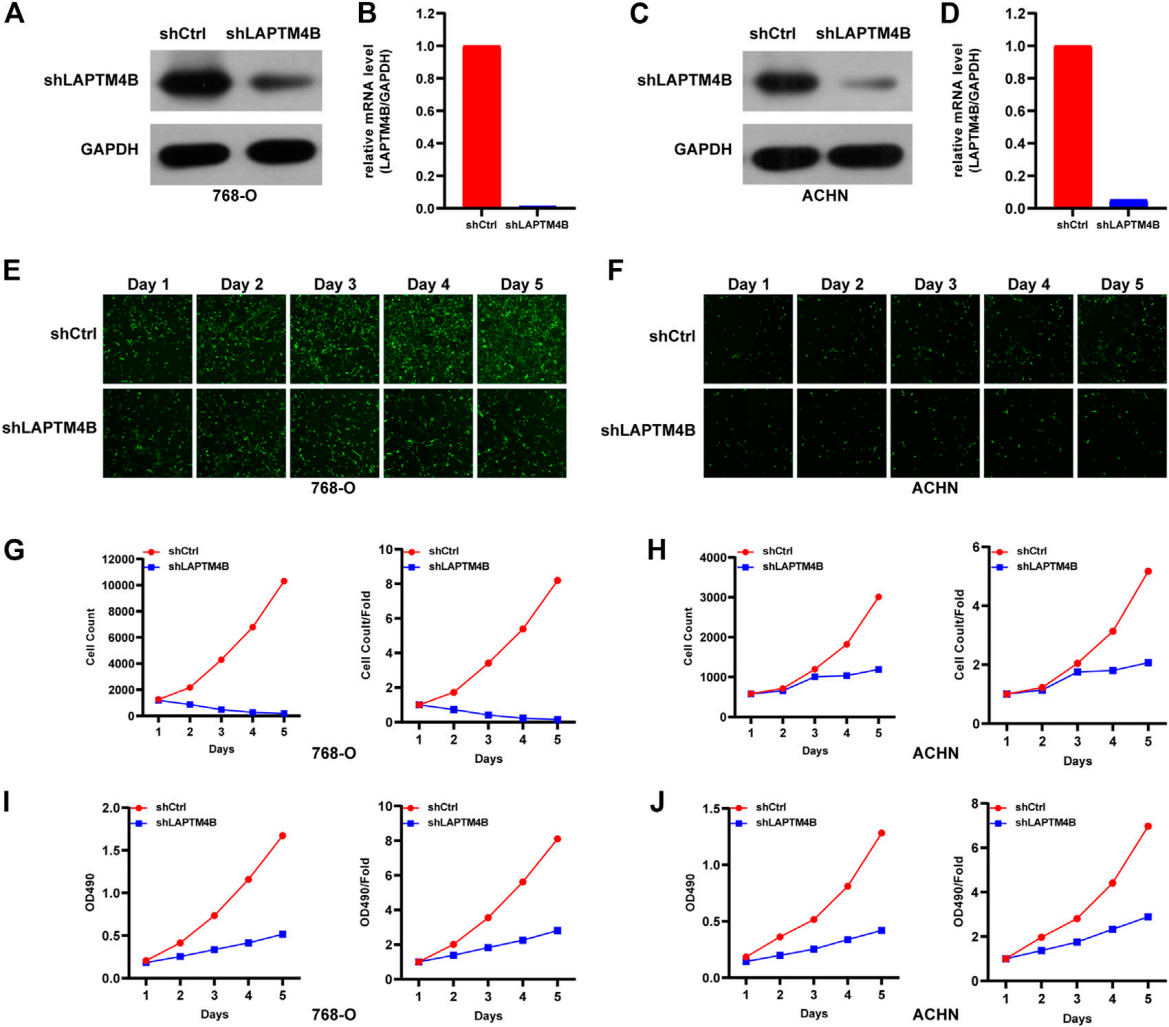
The role of LAPTM4B in ccRCC cell proliferation. **(A,B)** 786-O cells and **(C,D)** ACHN cells with LAPTM4B-shRNA lentivirus expressing either shCtrl and shLAPTM4B. GAPDH was used as the control. After LAPTM4B knockdown, **(E,G)** 786-O cells and **(F,H)** ACHN cells with GFP fluorescence detected by Celigo significantly decreased in 5 days. The numbers of ccRCC cells and proliferation rates were plotted for **(I)** 786-O cells and **(J)** ACHN cells.

To investigate the role of LAPTM4B in renal cell carcinoma, we performed experiments to detect the proliferation of ccRCC cells after LAPTM4B knockdown. Two cell lines with strong LAPTM4B expression (786-O and ACHN) were transfected with LAPTM4B-shRNA lentivirus. After 3 days of shRNA lentivirus infection, the expression of LAPTM4B was confirmed by qRT-PCR and western blotting assays; results showed the expression of LAPTM4B to obviously be decreased in shLAPTM4B cells than in shCtrl cells (Figures 7A, B). To investigate the effects of LAPTM4B on cell proliferation and viability, Celigo Image Cytometer analysis and MTT assay were used. Results showed that the counts of 786-O and ACHN cells were significantly lower in shLAPTM4B cells than in shCtrl cells in a time-dependent manner (Figures 7E–H). Furthermore, as shown in Figures 7I, J, the OD value of shLAPTM4B cells was lower than that of shCtrl cells. The data indicated that LAPTM4B contributes to the growth of renal cancer cells.

### 3.6 Knockdown of LAPTM4B induced apoptosis

**FIGURE 8:**
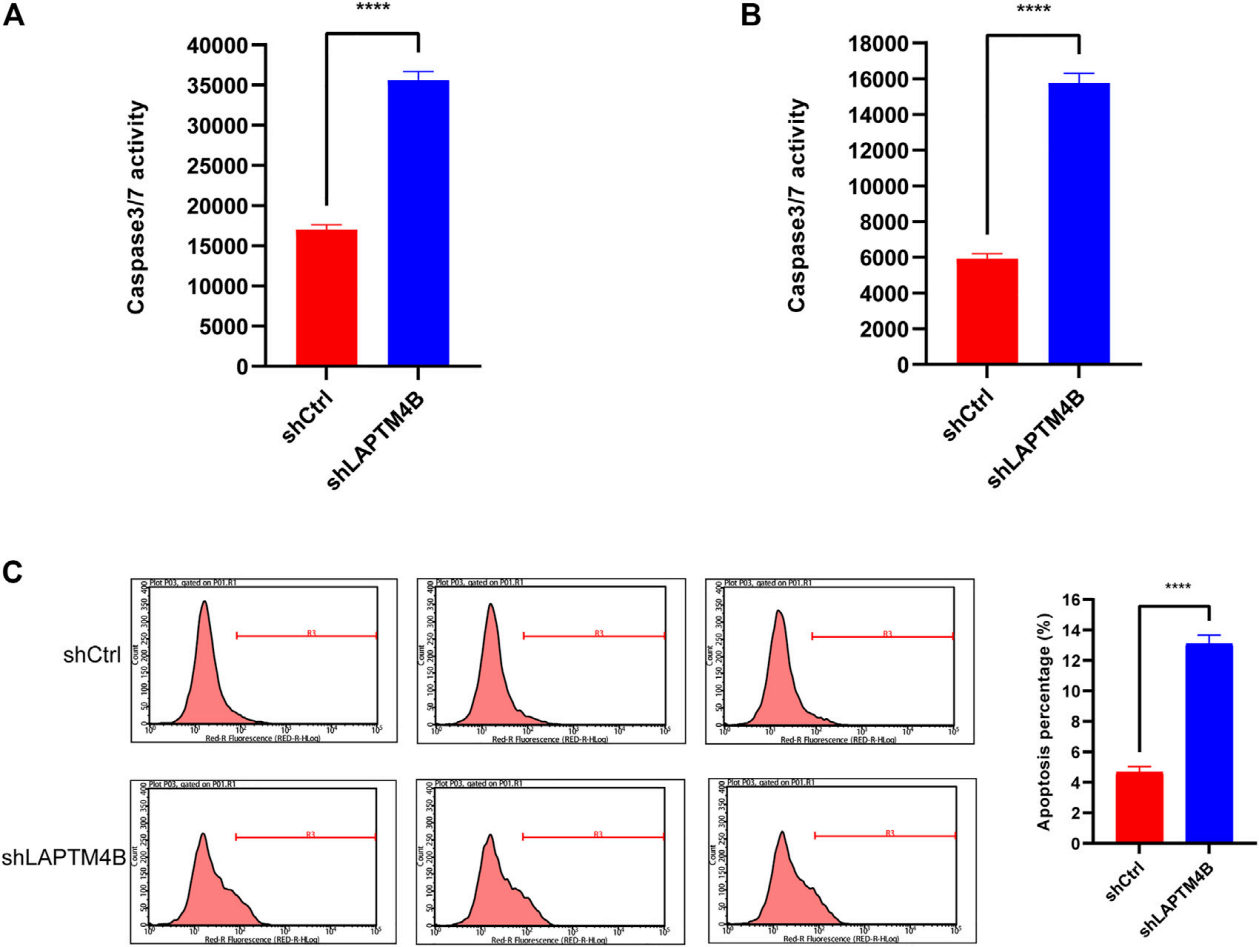
Knockdown of LAPTM4B induces apoptosis. **(A)** Caspase3/7 activity in 786-O. **(B)** Caspase3/7 activity in ACHN. **(C)** After the LAPTM4B knockdown, 786-O cells undergoing apoptosis increased significantly.

To evaluate whether LAPTM4B is involved in RCC apoptosis, as shown in Figures 8A, B, LAPTM4B was knocked out; caspase 3/4 activity in both types of cells was found to be significantly increased (*p* < 0.0001), as a result, compared to that in shCtrl cells. Knockout of LAPTM4B resulted in a considerably higher rate of apoptosis in 786-O cells than in shCtrl cells (*p* < 0.0001).

## 4 Discussion

In our study, we first identified that the LAPTM4B gene is related to RCC proliferation and metastasis. This ceramide-interacting protein, across quaternary membranes, can modulate the mTOR signaling pathway associated with autophagy (Dichlberger et al., 2021; Ji et al., 2022); LAPTM4B expression has been shown to be a poor prognostic marker in ovarian cancer (Yin et al., 2011). It would be worth noting that LAPTM4B-35, a protein encoded by LAPTM4B, is highly expressed in various solid tumors (Meng et al., 2016), and its overexpression is associated with poor prognosis in many malignancies, including ovarian, breast, cervical, and prostate cancers (Zhou et al., 2007; Qiao and Hu, 2015). Subsequently, we divided patients with ccRCC into two groups in the light of the different expression levels of LAPTM4B. Based on the DEGs between the two groups, we speculated that LAPTM4B might cause distinct differences in pathway activities. The immune differences between the two groups were compared, correlation between LAPTM4B and immune cells was also compared, and the activity differences in ccRCC tumor cells were compared using a single-cell study. Finally, we verified the correlation between LAPTM4B expression and tumor cell proliferation and metastasis using cell-based experiments.

Kidney cancer is one of the most rapidly developing tumors (Smittenaar et al., 2016). Although it is the second most common tumor in the urinary system, its mortality is the highest (Corgna et al., 2007; Capitanio et al., 2019; Siegel et al., 2022). Although both treatment and survival related to renal cell carcinoma have significantly improved, locally advanced disease and distant metastases are still diagnosed in many patients (Capitanio and Montorsi, 2016). Introduction of immune-related therapy in the treatment of renal cell carcinoma can alleviate tumorigenesis, although its effect is still not close to the ideal desired result, being closely related to the genetic heterogeneity within a tumor and the immunosuppressive effect of tumor microenvironment (Saeed et al., 2019; Incorvaia et al., 2021; Acosta et al., 2022). LAPTM4B mRNA exists in a variety of normal human tissues, with moderate expression in the kidney and high in the fetal kidney (Kasper et al., 2005). Our results (Figure 1C) showed that LAPTM4B is highly expressed in Fuhrman III/IV ccRCC tissues and are reduced in Fuhrman I/II ccRCC tissues, suggesting the phenomenon to possibly be related to the heterogeneity of renal cancer. This heterogeneity affects the development of the disease and the adverse effects of treatment, thereby posing a significant threat to the prognosis and survival of patients, besides being an additional economic burden (Usher-Smith et al., 2020). Therefore, investigation of the mechanism underlying the occurrence and development of renal cancer is imperative in order to provide effective targeted therapy.

Autophagy is could safeguard the cell and body homeostasis, mutations in autophagy-related processes always lead to severe human pathologies (Klionsky et al., 2021). Recent studies have shown that autophagy is closely associated with kidney-related diseases (Choi, 2020; Wang et al., 2020). In our functional enrichment results (Figures 2, 3), DEGs in different groups were involved in EGFR, mTOR, PIK3, JAK/STAT3, p53, and other autophagy-related or cell proliferation-related pathways. LAPTM4B can enhance and prolong EGFR signaling by blocking active EGFR intraluminal sorting and lysosomal degradation. Therefore, LAPTM4B may be associated with autophagy through the EGFR signaling pathway (Wu and Zhang, 2020; Ji et al., 2022). Inactive EGFR and LAPTM4B can be mutually stable, and the decrease of either will cause the inhibition of the autophagy process. This may be due to inactive EGFR and LAPTM4 B’s interaction with the recruitment of extracellular subcomplexes containing Sec5, which facilitate EGFR’s association with the autophagy inhibitor Rubicon (Tan et al., 2015b). LAPTM4B can also inhibit autophagy by blocking EGF-stimulated enzyme-somal degradation to enhance EGFR signaling (Tan et al., 2015a). Inhibiting the PI3K/AKT/mTOR signaling pathway not only enhances the active level of autophagy but also plays a vital role in inducing apoptosis in RCC (Liu et al., 2021; Tang et al., 2021). p53 is a tumor suppressor gene that is closely related to tumorigenesis. Its product can induce cell cycle arrest or apoptosis reactive to DNA damage, thereby keeping the genetic stability of the organism (Steele et al., 1998; Bykov et al., 2018). An important relationship exists between autophagy and p53 expression. Autophagy inhibits p53 while p53 activates autophagy (White, 2016). Autophagy can directly inhibit cell growth by targeting the degradation of p53 (Smith and Macleod, 2019). The IL-6/JAK/STAT3 pathway is abnormally enhanced in many tumors, often indicating poor prognosis. In the tumor microenvironment, IL-6/JAK/STAT3 signaling pathway drives malignant cell proliferation, survival, invasion, and metastasis while strongly inhibiting the antitumor immune response (Johnson et al., 2018). Changes in STAT3 expression are accompanied by abnormal autophagy, which may affect each other during tumor development (Xu et al., 2022). Chemical inhibition of STAT3 can induce autophagy, and high STAT3 expression can strongly inhibit autophagy. In addition, STAT3 phosphorylation can negatively regulate autophagy (Siegelin et al., 2010; Liu et al., 2018).

In addition to regulating cell proliferation and death pathways, autophagy acts as a crucial element affecting infection, inflammation, and immune (Deretic et al., 2013). Autophagy can regulate the function of immune and the production of cytokines to significantly control the immune response, which is an essential target of immunotherapy. Autophagy may also be a favorable factor for tumor cells to get out of immune surveillance, which is one of the factors contributing to intrinsic resistance to tumor immunotherapy (Jiang et al., 2019). Kidney cancer is a tumor with solid heterogeneity, tumor antigenic changes, and a complex immune microenvironment for tumor immune escape. MHC and VEGF expression can promote immune surveillance of renal cancer (Jian et al., 2021). The MHC complex and VEGF pathways were significantly enriched in the GSVA enrichment results of DEGs and in the two groups (Figures 2, 3). Under normal circumstances, CD8 + T-cell infiltration is a good prognostic factor in patients with cancer; however, it lacks a disadvantage in patients with renal cancer, which might explain why kidney cancer is sensitive to immunotherapy (Kim et al., 2021). In the results of immune infiltration (Figures 4, 5), we observed that expression of LAPTM4B is positively correlated with CD8 T cells, and is lower in high-grade renal cancer than in early-stage renal cancer, possibly explaining the poor response to treatment in advanced renal cancer. The number of M2 macrophages was significantly higher in the high group than the other. IL-10 and TGF-β secreted by M2 macrophages can inhibit the activation of CTL and NK cells, reduce their killing effect on tumor cells, and indirectly promote tumor proliferation (Yunna et al., 2020). Simultaneously, M2 macrophages are known to promote the formation of new tumor blood vessels and indirectly promote the infiltration and metastasis of tumor cells (Wang et al., 2017; Guo et al., 2021). M2 macrophages are essential in the occurrence and development of ccRCC. M2 macrophages can enhance the proliferation, migration, invasion, and EMT of ccRCC cells in an Akt-dependent manner by secreting CXCL13 (Xie et al., 2021). The negative correlation (Table 1) between LAPTM4B and most immune checkpoints partially explained the insensitivity of ccRCC to immunotherapy, this evidence suggested that LAPTM4B may promote the growth and metastasis of renal cancer by boosting the immune escape of tumor cells. Existing literature has proved that LAPTM4B can promote cell proliferation and growth, metastasis and invasion, and inhibit cell apoptosis, which is consistent with our experimental results (Figures 7, 8) (Meng et al., 2016). LAPTM4B may influence apoptosis through its expression level and ceramide compartmentalization. The cells with high LAPTM4B expression showed increased ceramide clearance in the late endonuclease body, which generated the cells sensitive to ceramide-induced apoptosis. Meanwhile, the late endosome membrane is stable, and the cells are insensitive to lysosomal-mediated death. On the other hand, cells with low LAPTM4B expression sequestrated ceramide in late endosomes, protecting cells from late endosomal ceramide toxicity, but sensitivities cells to lysosomal mediated death (Blom et al., 2015).

By evaluating the pathway activity in single cells, we found no significant difference in the expression of autophagy activity across all cell types. In contrast, lysosomal activity was more evident in immature macrophages and monocytes, which could be in connection with the clear effect of immune cells. However, LAPTM4B expression was more evident in stem and endothelial cells, but not in macrophages, which is also a limitation of this study. This phenomenon could be attributed to our single-cell data being from a database, the sample size being small, and presence of experimental error due to the lack of multi-sample verification. To circumvent this limitation, the results may be verified using large-sized sequencing samples in clinical practice, in order to improve the mechanism of LAPTM4B in renal cancer. In conclusion, LAPTM4B is an adverse factor in the development of renal cancer. Targeted therapy for LAPTM4B and early intervention for patients with RCC can help improve their survival status and quality of life.

## Data Availability

The datasets presented in this study can be found in online repositories. The names of the repository/repositories and accession number(s) can be found in the article/supplementary material.
